# A palmitoyltransferase *Approximated* gene *Bm‐app* regulates wing development in *Bombyx mori*

**DOI:** 10.1111/1744-7917.12629

**Published:** 2018-08-23

**Authors:** Ye Yu, Xiao‐Jing Liu, Xiao Ma, Zhong‐Jie Zhang, Tai‐Chu Wang, Fan Sun, Cheng‐Xiang Hou, Mu‐Wang Li

**Affiliations:** ^1^ School of Biotechnology Jiangsu University of Science and Technology Zhenjiang Jiangsu China; ^2^ Sericultural Research Institute Chinese Academy of Agricultural Sciences Zhenjiang Jiangsu China; ^3^ Sericultural Research Institute Anhui Academy of Agricultural Sciences Hefei China

**Keywords:** *Bombyx mori*, CRISPR/Cas9, minute wing, palmitoyltransferase, promoter

## Abstract

The silkworm *Bombyx mori* is an important lepidopteran model insect in which many kinds of natural mutants have been identified. However, molecular mechanisms of most of these mutants remain to be explored. Here we report the identification of a gene *Bm‐app* is responsible for the silkworm minute wing (*mw*) mutation which exhibits exceedingly small wings during pupal and adult stages. Compared with the wild type silkworm, relative messenger RNA expression of *Bm‐app* is significantly decreased in the u11 mutant strain which shows *mw* phenotype. A 10 bp insertion in the putative promoter region of the *Bm‐app* gene in *mw* mutant strain was identified and the dual luciferase assay revealed that this insertion decreased *Bm‐app* promoter activity. Furthermore, clustered regularly interspaced short palindromic repeats/RNA‐guided Cas9 nucleases‐mediated depletion of the *Bm‐app* induced similar wing defects which appeared in the *mw* mutant, demonstrating that *Bm‐app* controls wing development in *B. mori*. *Bm‐app* encodes a palmitoyltransferase and is responsible for the palmitoylation of selected cytoplasmic proteins, indicating that it is required for cell mitosis and growth during wing development. We also discuss the possibility that *Bm‐app* regulates wing development through the Hippo signaling pathway in *B. mori*.

## Introduction

Insects are the only winged invertebrates among arthropods and their flight ability is a key factor underlying their expansion, invasion, proliferation and reproduction (Lewin, 1985; Engel & Grimaldi, 2004). The wing has become an important model system in evolutionary biology, ecology and physiology (Zera & Denno, 1997; McCulloch *et al*., 2009). The silkworm *Bombyx mori* is an important lepidopteran model organism and more than 400 silkworm mutants have been identified in nature (Goldsmith *et al*., 2005). Among these mutants, multiple studies have been performed to investigate *B. mori* wing‐related mutations since they are ideal genetic materials for exploring the molecular basis of insect wing development. The wing‐related mutation in *B. mori* shows high diversity such as crayfish pupa (*cf*) (Tong *et al*., 2015). *wingless locus flugellos* (*fl*) (Fujiwara & Hojyo, 1997), *winglet* (*rw*) and *minute wing* (*mw*) (Goldsmith *et al*., 2005). The molecular mechanisms underlying *fl* (Fujiwara & Hojyo, 1997; Matsunaga & Fujiwara, 2002) and *non‐lepis wing* (*nlw*) (Zhou *et al*., 2004, 2006) mutations have been reported. However, most of these mutations remain to be functionally analyzed and little is known about the molecular mechanism generating these diversities.

**Figure 1:**
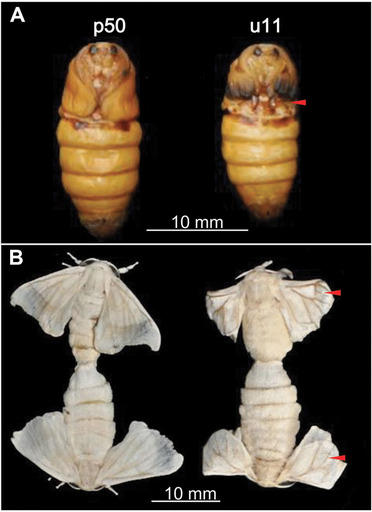
Phenotypes of wild‐type (p50) and *mw* mutant (u11) strains. Pupal and moth stages of the wild‐type (A) and *mw* mutant (B) are shown. Red arrows indicate smaller and slightly curled wings in u11 mutant moths.

Silkworm *mw* mutants show small and curly wings during pupal and adult stages compared with wild type animals (Fig. 1). In the previous study, we constructed a simple linkage map of *mw* in *B. mori* (Ma *et al*., 2013). However, it remains to be clarified which gene causes *mw* phenotype due to difficulty in positional cloning and lack of genetic tools. Recently, genome‐editing tools such as ZFN (zinc‐finger nucleases), TALEN (transcription‐activator like effector nucleases) and the CRISPR/Cas9 (clustered regularly interspaced short palindromic repeats/RNA‐guided Cas9 nucleases) systems have been applied in functional gene analysis in different organisms (Hruscha *et al*., 2013; Wang *et al*., 2013). In *B. mori*, these techniques have also been successfully established (Wang *et al*., 2013; Li *et al*., 2015; Xu *et al*., 2017; Zeng *et al*., 2017), providing effective loss‐of‐function toolkits to explore the mechanisms for silkworm natural mutants such as *mw*.

In the current study, we describe identification of a palmitoyltransferase *Approximated* gene *Bm‐app* that is responsible for *mw* phenotype. Positional cloning revealed that *Bm‐app* locates in a 275 kb region on chromosome 22 which contains 15 *mw* candidate genes including *Bm‐app*. We generated somatic mutagenesis targeting *Bm‐app* by direct injection of Cas9 messenger RNA (mRNA) and sequence‐specific single‐guide RNAs (sgRNAs), resulting in the shrinkage of wings during the pupal and adult stages which are similar to *mw* natural mutants of the u11 strain. Further analysis reveals that a 10 bp insertion in the promoter region of *Bm‐app* gene resulted in low level expression. These findings reveal that *Bm‐app* regulates wing development in the silkworm.

## Materials and methods

### Silkworm strains

A bivoltine silkworm strain, u11, which shows minute wing (*mw*) phenotype, was used as the natural mutant strain. Another bivoltine silkworm strain, p50, which shows normal wings, was used as wild type animals for the back‐crossing strategy. A multivoltine silkworm strain, Nistari, was used in generating CRISPR/Cas9‐mediated mutagenesis and subsequent experiments. All silkworm strains were preserved in the Sericultural Research Institute, Chinese Academy of Agricultural Sciences, and larvae were fed with fresh mulberry leaves at 25 °C under standard conditions (Tan *et al*., 2005).

The BmN cell line originating from the *B. mori* ovary was used (Pan *et al*., 2010). The cells were maintained in TC‐100 medium (AppliChem, Gatersleben, Germany) containing 10% fetal bovine serum in 25 cm^2^ Petri dishes at 27 °C. The mammalian HEK293T cell line was maintained at 37 °C under 5% CO_2_ in Dulbecco's modified Eagle's medium high glucose medium (Gibco, Grand Island, NY, USA) containing 10% fetal bovine serum (Gibco).

### Positional cloning of mw candidate genes

Since *mw* is a recessive single locus, crosses based on a single‐pair backcross model were designed (Yutaka *et al*., 2005). The u11 mutant strain was crossed with the wild‐type p50 strain to prepare reciprocal populations. The resulting female F_1_ and male F_1_ crosses were backcrossed with *mw* to generate female backcross populations BC_1_F and male backcross populations BC_1_M. Due to the lack of crossing over between chromosomes Z and W of silkworm females, reciprocal backcrossed BC_1_F progeny were used for linkage analysis and mapping of the *Bm‐mw* gene in chromosome 22 using backcrossed BC_1_M. The sequence‐tagged site (STS) molecular marker linkage was used to make the linkage map. Linkage maps were generated by MAPMAKER 3.0 using the Kosambi function, as described elsewhere (Lander *et al*., 1987).

### RNA isolation and cDNA synthesis

Total RNA was isolated from the wing disc on the 6th day of the 5th larval instar (wandering stage) using TRIzol reagent (Invitrogen, Carlsbad, CA, USA) according to the manufacturer's instructions and subsequently was treated with DNase I (Invitrogen) to remove genomic DNA. To synthesise cDNA, 1 μg of total RNA was used with the RevertAid First Strand cDNA Synthesis Kit (Fermentas, Vilnius, Lithuania).

### Quantitative real‐time PCR (qPCR) analysis

qPCR was performed to analyze the relative mRNA expression levels of selected genes. cDNA synthesized from total RNA isolated from the wing disc at the wandering stage was used as the template. The PCR conditions were as follows: initial incubation at 95 °C for 1 min, 40 cycles of 95 °C for 15 s, and 60 °C for 1 min. The cycling conditions were as follows: initial incubation at 95 °C for 10 min, 45 cycles of 95 °C for 15 s, and 60 °C for 1 min. The primers that were used in qPCR to investigate the mRNA expression levels of the genes of interest are listed in Supplementary Material Table S1. Another primer pair, RP49‐F and RP49‐R (Table S1), which amplifies a 136 bp fragment from the *B. mori* ribosomal protein 49, was used as an internal control (Tan *et al*., 2013). The data shown are mean ± SEM (*n* = 3). The asterisks indicate the significant differences with a two‐tailed *t*‐test: ^*^ *P* < 0.05; ^**^ *P* < 0.01; ^***^ *P* < 0.0001.

### Preparation and microinjection of Cas9/sgRNA

The Cas9‐sgRNA system was used to target the candidate gene *Bm‐app*. Site one (S1) and site two (S2) were identified by screening the genomic sequence of *Bm‐app mw* following the 5ʹ‐GG‐N18‐NGG‐3ʹ rule (Hruscha, 2013; Hwang, 2013). The sgRNAs were prepared using a MAXIscrip^R^ T7 kit (Ambion, Austin, TX, USA), according to the manufacturer's instructions. Cas9 mRNA was synthesized using a mMESSAGE mMACHINE^R^ T7 kit (Ambion), according to the manufacturer's instructions, as previously reported (Wang *et al*., 2013).

Fertilized eggs were prepared as previously described (Zhang *et al*., 2015) and micro‐injected within 3 h after oviposition. A mixture solution of Cas9 mRNA (300 ng/mL), sgRNA‐1 (150 ng/mL) and sgRNA‐2 (150 ng/mL) were injected into 480 embryos. Injection with an equal amount of phosphate‐buffered saline (PBS) was performed as a control. The injected eggs were incubated at 25 °C in a humidified chamber for 10–12 days until larval hatching (Tan *et al*., 2013).

### Genomic DNA extraction and mutagenesis analysis

Genomic PCR, followed by sequencing, was carried out to identify the *Bm‐app* mutant alleles induced by Cas9/sgRNA injection. Genomic DNA was extracted from the heterozygote with the DNA extraction buffer (1:1:2:2.5 ratio of 10% sodium dodecyl sulfate to 5 mol/L NaCl to 100 mmol/L ethylenediaminetetraacetic acid to 500 mmol/L Tris‐HCl, pH = 8), incubated with proteinase K and purified via standard phenol : chloroform extraction and isopropanol precipitation extraction, followed by RNaseA treatment. The PCR conditions were as follows: 98 °C for 2 min, 35 cycles of 94 °C for 10 s, 55 °C for 30 s, and 72 °C for 40 s, followed by a final extension period of 72 °C for 10 min. The PCR products were cloned into pJET1.2 vectors (Fermentas) and directly sequenced. The primer sequences are listed Table S1.

### Plasmid construction and cell transfection

A plasmid *PXL‐IE1DsRed2‐EGFP* was used to construct *PXL‐IE1DsRed2*‐u11‐EGFP (*PXL*‐*u11‐EGFP*) or *PXL‐IE1DsRed2*‐p50‐EGFP (*PXL*‐*p50‐EGFP*) plasmid as previously described (Xu *et al*., 2014). In the *PXL‐u11‐EGFP* plasmid, the enhanced green fluorescent protein (EGFP) reporter gene was driven by a putative promoter sequence of *Bm‐app* cloned from the u11 strain. A control plasmid *PXL‐p50‐EGFP*, was also constructed in which the EGFP reporter gene was driven by a putative promoter sequence of *Bm‐app* cloned from the p50 strain. Additionally, there was a red fluorescent protein DsRed2 expression cassette driven by the IE1 promoter in three vectors as the selecting marker. The putative promoter sequence was PCR amplified and introduced into *KpnI* and *ApaI* restriction sites of *PXLBacII‐IE1DsRed2‐EGFP* to generate *PXL‐u11*‐*EGFP* or *PXL*‐*p50*‐*EGFP*, respectively.

Two hundred nanograms of each plasmid were transfected into BmN cells in a 24‐well cell culture plate in duplicate using lipofectamine reagent (Invitrogen), following the manufacturer's instruction. EGFP fluorescence was investigated 72 h after transfection under a fluorescence stereomicroscope (Nikon AZ100, Tokyo, Japan).

### Dual luciferase reporter (DLR) assay

For high transfection efficiency and low background expression of *Bm‐app*, the mammalian HEK293T cell line was used for the DLR assay. Different genomic fragments containing potential promoter sequences of 2 kb, 1.5 kb, 1 kb and 0.5 kb, were cloned and inserted into the pGL3‐promoter plasmid separately (Promega, Madison, WI, USA) between the firefly luciferase open reading frame (ORF) and SV40 poly(A). The HEK293T cells were transfected with 5 ng of the pGL3 reporter plasmid, 5 ng of the pRL‐TK control plasmid mixed with 0.5 µL Lipofectamine 2000 Transfection Reagent (Gibco) in each well of a 96‐well plate. Equal pGL3‐Basic plasmid and pRL‐TK control plasmid transfected as negative control. The DLR Assay (Promega) was performed according to the manufacturer's protocol 48 h after transfection. The experiments were performed in triplicate with three technical repeats. The mean of the relative luciferase expression ratio (firefly luciferase/ renilla luciferase) of the control was set to 1.

## Results

### Identification of the mw candidate gene

**Figure 2:**
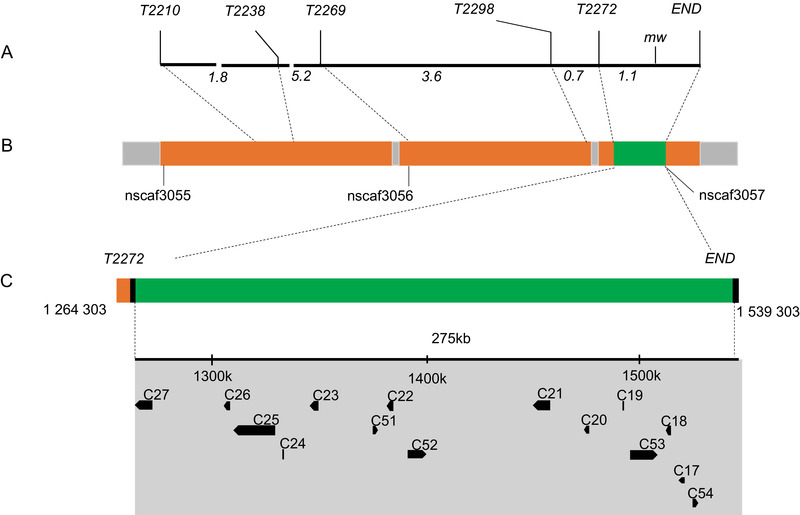
*Bm‐mw* mapping on *Bombyx mori* chromosome 22 and identification of candidate genes. (A) A linkage map based on BC1M genotyping was constructed for group 22. Five sequence‐tagged site (STS) markers and the *Bm‐mw* locus are shown above and distances between loci (cM) are shown below. (B) Scaffold map of chromosome 22, based on data from the SilkDB database. Orange boxes represent the assembled scaffolds, the names of which are given beneath. The STS markers used for genotyping are localized on the scaffold positions, as indicated by the dotted lines. The green box shows the candidate region linked to the *mw* locus. The enlarged candidate region is shown beneath along with the position on nscaf3056 of T2272 and terminal, which fix the candidate region. (C) Above is shown the coordinates of nscaf3056. The gray area represents the candidate region, which includes 15 predicted genes according to SilkDB. The directions of the transcription of these genes are represented by arrowheads. For convenience, they are named C17 to C27, C51 to C54 based on their positions. Detailed information for these 15 genes is given in Table S2 in the supplementary material.

**Figure 3:**
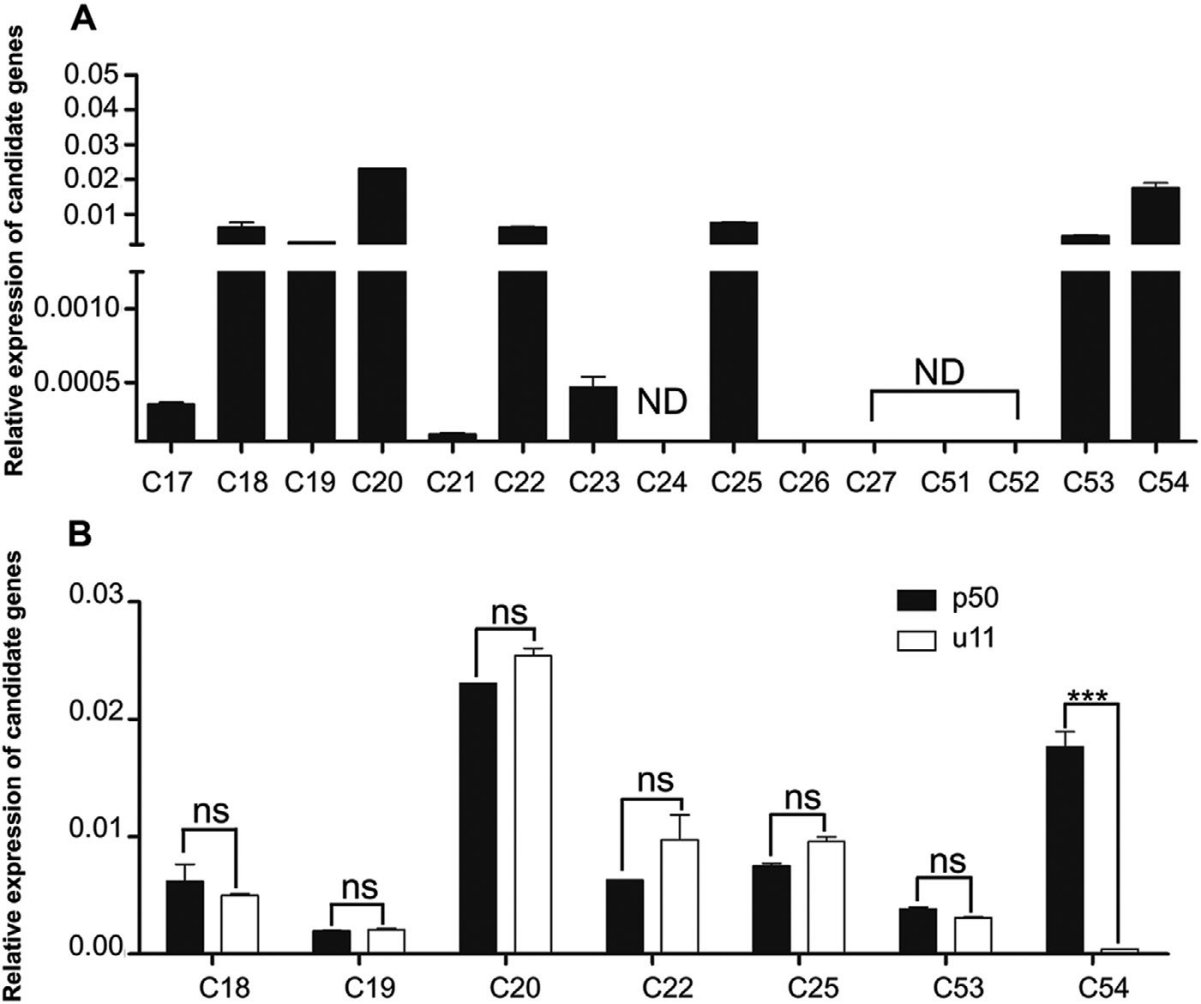
Quantitative real‐time polymerase chain reaction analysis of the minute wing candidate genes. (A) Expression patterns of 15 candidate genes in the wing disc of wandering stage larvae. (B) Expression of seven genes between p50 and u11 strains. The asterisks (^***^) indicate significant differences (*P* < 0.0001) compared with the relevant control with a two‐tailed *t*‐test. Error bars depict ± SEM. ND, not determined. NS, no significance.

In the previous study, we constructed a genetic linkage map between *Bm‐mw* gene and STS markers in *B. mori* (Ma *et al*., 2013). However, it remains to be clarified which gene causes the *mw* phenotype. On this basis, we used five STS markers (T2238, T2210, T2269, T2298 and T2272) which were searched at various positions along the nucleotide sequence of the scaffold nscaf3056 on chromosome 22 (Fig. 2A). PCR‐based DNA fragment amplification and the restriction fragment length polymorphism method were used to map the *mw* locus to the genomic DNA sequence and revealed a polymorphism between u11 and p50 strains. The genome distance was 275 kb between T2272 which was the closest marker to *mw* and the end of the chromosome. Since no other STS markers were found in this region, the *mw* locus was finally mapped to the 275 kb region between marker T2272 and the end of nscaf3056 (Fig. 2). Within this region, 15 candidate genes were identified according to the SilkDB database (Xia *et al*., 2004) (Fig. 2 and Table S2). We then performed qPCR analysis to investigate their relative mRNA expressions in the larval wing disc of the p50 strain (Fig. 3). Eight genes (C17, C21, C23, C24, C26, C27, C51 and C52) showed low or no signals in the wing disc and thus could be excluded (Fig. 3A). For the rest of the seven genes (C18, C19, C20, C22, C25, C53 and C54) which showed relatively high mRNA expressions in the wing disc (Fig. 3A), we further compared their relative mRNA expressions in the wing disc between p50 and u11 strains. As a result, only C54 showed significant difference and the mRNA expression level of C54 was much lower in the u11 strain compared with the p50 strain (Fig. 3B). These data suggested that C54 was the most possible candidate gene for *mw* phenotype.

### CRISPR/Cas9‐mediated mutagenesis of C54 (Bm‐app)

**Figure 4:**
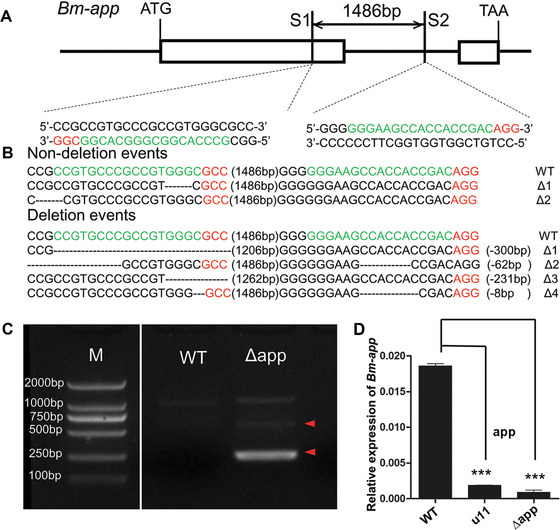
Schematic diagram of single‐guide RNA (sgRNA) targeting sites. (A) Schematic diagram of sgRNA‐targeting sites. The boxes indicate the two exons of *Bm‐app*, and the black line represents the gene locus. The sgRNA targeting sites, S1 and S2, are located on the sense strand of exon‐1 and the sense strand of intron, respectively. The protospacer adjacent motif (PAM) sequence is shown in red. (B) Various types of mutagenesis induced by Cas9/sgRNA injection. The numbers in brackets in the middle of each sequence refer to the 1486 bp‐long interspace fragment that was found between the S1 and S2 sites. The red sequence indicates the PAM sequence. (C) Genomic polymerase chain reaction analyses revealed deletion mutation events in the mutants. The red arrowhead indicates the deleted region. (D) The transcript level of *Bm‐app* is down‐regulated significantly in *Δapp* animals. The asterisks (^***^) indicate the significant differences (*P* < 0.0001) compared with the relevant control with a two‐tailed *t*‐test. Error bars depict ± SEM.

To further verify whether C54 was responsible for the *mw* phenotype, CRISPR/Cas9‐mediated, targeted mutagenesis analysis was performed. The candidate gene C54 is designated as *Bm‐app* which encodes a palmitoyltransferase *Approximated* gene. Genomic structure of *Bm‐app* showed it has two exons with one intron (Fig. 4A) and encodes a protein of 110 amino acid residues and spanned approximately 2.8 kb on chromosome 22. Screening of the genomic sequence of *Bm‐app* according to the GGN18NGG rule identified two sgRNA target sites of site 1 (S1) and site 2 (S2) (Fig. 4A). These two sites located on exon 1 and intron 1, respectively, and the fragment spanning the two sites was 1486 bp in length (Fig. 4A).

**Figure 5:**
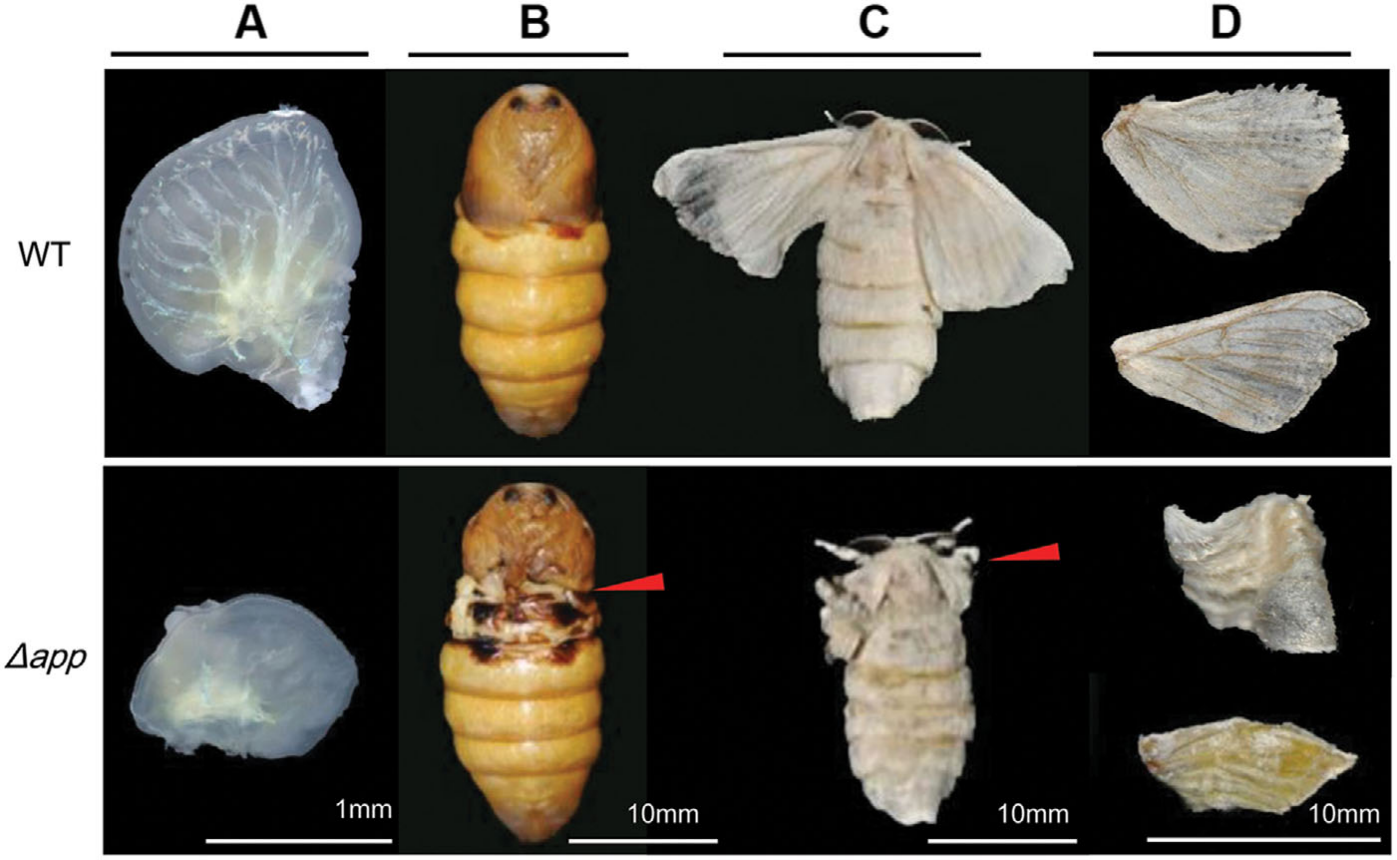
Mutants with abnormal wings induced by Cas9/single‐guide RNA injection. The mutants showed small and curly wing discs during the larval stage (A) and small wings during pupal (B) and adult stages (C, D) The red arrows indicated abnormal position.

The sgRNAs that were synthesized *in vitro* were mixed with Cas9 mRNA and injected into preblastoderm embryos of the Nistari strain. In total, 480 eggs were injected with the Cas9/sgRNA mixture at a concentration of 300 ng/µL Cas9 mRNA and 300 ng/µL of each sgRNA. Among injected embryos, 71.3% (*n = *342) hatched and 283 individuals survived to adult stages. Of the surviving moths, 86.6% (*n = *245) showed defects in wings. In contrast, 480 eggs were injected with PBS and 78.1% (*n = *375) hatched, 312 individuals survived to adult stages and no wing defects appeared in moths (Fig. 5). The majority of the mutant pupae exhibited minute wings not covering the meta‐thorax and moths exhibited malformed, curly wings, and the forewings did not cover the hind wings (Fig. 5). To confirm that the wing developmental defects described above were due to genomic mutagenesis induced by Cas9/sgRNA injection, genomic DNA was extracted from five randomly selected moths with *mw* phenotype. The fragments spanning one or both target sites were then PCR‐amplified and sequenced. PCR‐based analysis revealed mixed bands appeared in mutants (Fig. 4C) and both insertion and deletion mutations were detected (Fig. 4B). The transcript level of *Bm‐app* was also down‐regulated significantly in mutants (Fig. 4D). These data proved successful targeted mutagenesis occurred at the *Bm‐app* locus.

### Identification and validation of Bm‐app cis‐regulatory elements in the u11 mutants

No difference was detected in coding sequence of *Bm‐app* between p50 and u11 strains. However, relative mRNA expression of *Bm‐app* showed significant down‐regulation in the u11 strain (Fig. 3B), indicating that *cis*‐regulatory elements might be responsible for *Bm‐app* down‐regulation. We thus separately PCR‐amplified 2 kb putative promoter sequences of *Bm‐app* in p50 and u11 strains. Promoter prediction (http://www.fruitfly.org/seq_tools/promoter.html) revealed that there were six putative promoter sequences with score cutoff of 0.80 in the p50 strain (Supplementary sequence). The sequence in the u11 strain was identical, except there was a 10 bp insertion (ACATAGTAGT) which was located in one of the putative promoter sequences (Supplementary sequence).

**Figure 6:**
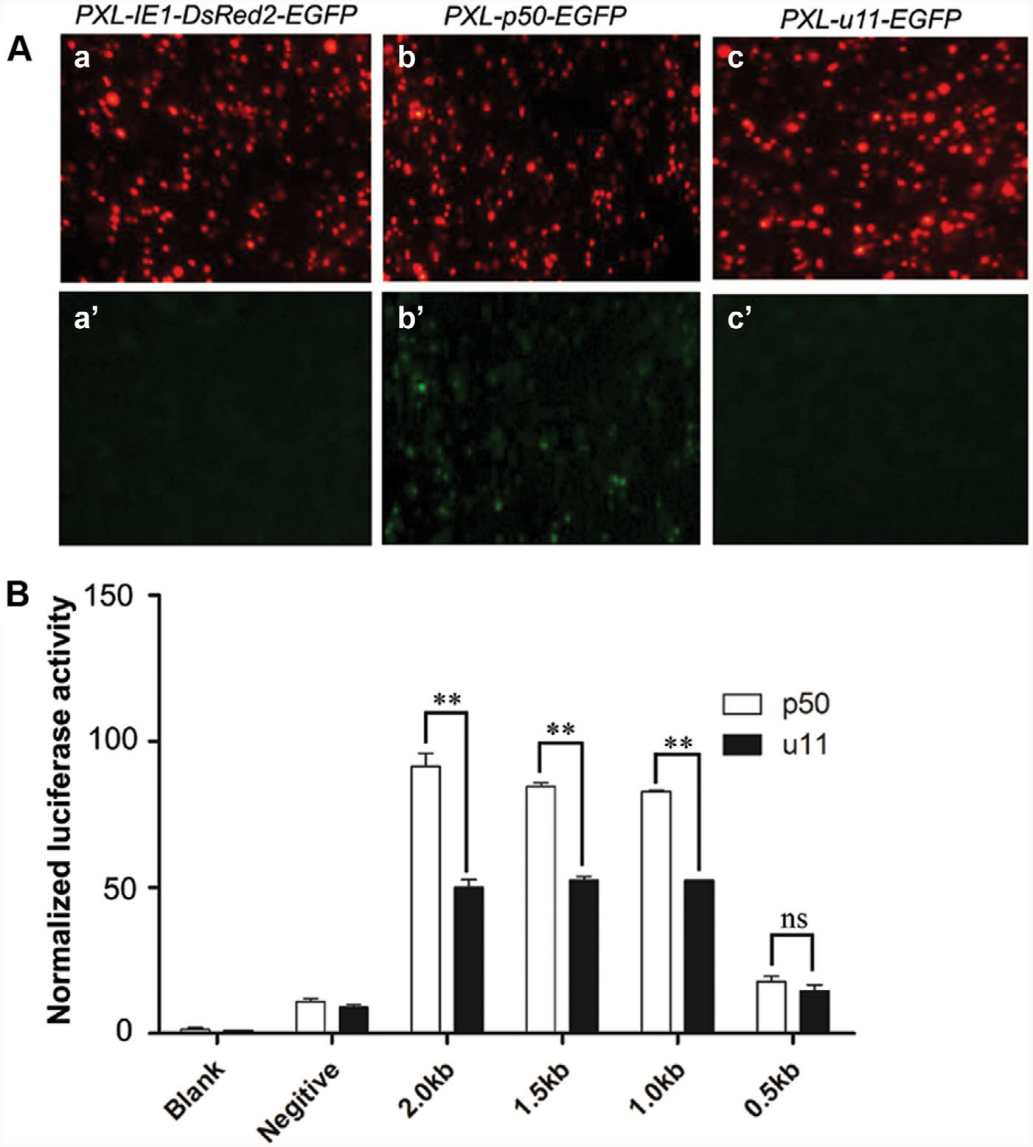
Promoter verification in BmN and HK293T cell lines. (A) Putative 2 kb promoter sequences of *Bm‐app* in p50 and u11 strains were sub‐cloned into *PXL‐p50‐EGFP* or *PXL‐u11‐EGFP* plasmid separately to perform cell transfection by using the BmN cell lines. *PXL‐IE1Dsred2‐EGFP* was used as a control. All three transfected plasmids could drive DsRed2 expression (a–c) while only *PXL‐p50‐EGFP* could drive enhanced green fluorescent protein (EGFP) expression (a′–c′). (B) *Bm‐app* promoter activity was analyzed in p50 and u11stains by measuring the Firefly/Renilla ratio. Blank, non‐transfected cells; negative, pGL3‐Basic‐transfected cells; tested, cells transfected with *Bm‐app* promoter‐2.0 kb (2.0 kb), *Bm‐app* promoter‐1.5kb (1.5 kb), *Bm‐app* promoter‐1.0kb (1.0 kb) and *Bm‐app* promoter‐0.5kb (0.5 kb) in p50 and u11 stains. All data are representative of three independent experiments and expressed as the mean ± SEM. The asterisks (^**^) indicate significant differences (^**^ *P* < 0.01) compared with the p50 stain.

To verify if this insertion was responsible for *Bm‐app* promoter activities, these 2 kb putative promoter sequences of *Bm‐app* in p50 and u11 strains were sub‐cloned into *PXL‐p50‐EGFP* or *PXL‐u11‐EGFP* plasmid separately to perform cell transfection by using the BmN cell lines. The results showed that the promoter sequence in *PXL‐p50‐EGFP* could drive EGFP expression while that in *PXL‐u11‐EGFP* did not (Fig. 6A). Further, different lengths of 0.5 kb, 1 kb, 1.5 kb and 2 kb upstream sequences were PCR‐amplified from p50 and u11 strains separately and sub‐cloned into pGL3‐promoter plasmid to perform DLR Assay in HK293T cell lines (Fig. 6). The result showed that there was significant difference in DLR activities between p50 and u11 strains when 1 kb, 1.5 kb or 2 kb sequences were subjected (Fig. 6B). The DLR activities decreased significantly when 0.5 kb sequence was applied (Fig. 6B). These data revealed that 10 bp insertion in u11 strain significantly decreased promoter activity, suggesting that it contributed to *mw* phenotype.

### Transcription levels of genes in the Hippo pathway affected by Bm‐app down‐regulation

**Figure 7:**
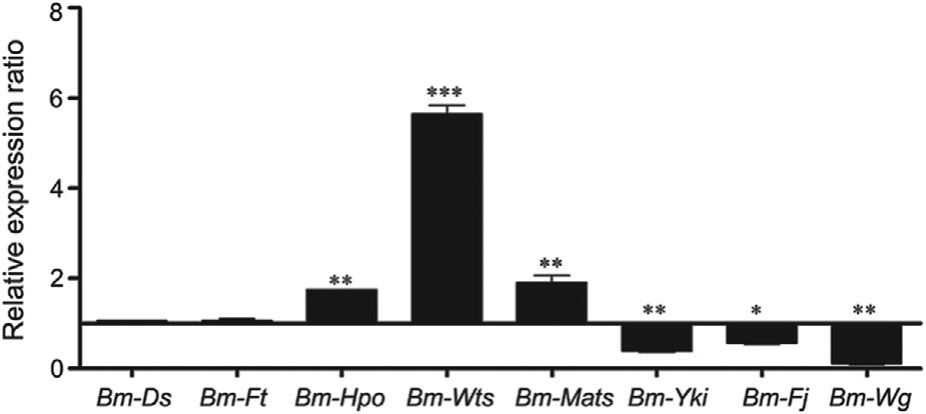
*Bm‐app* mutagenesis affected genes expression of the Hippo pathway in *Bombyx mori*. Relative messenger RNA expressions of genes in the Hippo signalling pathway were determined by quantitative real‐time polymerase chain reaction in wild‐type (WT) and *Δapp* silkworms. The asterisks (^*^ or ^**^ or ^***^) indicate significant differences (^*^ *P* < 0.05; ^**^ *P* < 0.01; ^***^ *P* < 0.001) compared with the WT. Error bars depict ± SEM.

In *Drosophila*, App can bind both Dachsous (Ds) and Fat (Ft), the important components of the Hippo pathway (Mao *et al*., 2006; Zhang *et al*., 2016). App promotes the activity of Ds by simultaneously repressing Ft via post‐translational modification and recruiting Ds to the apical junctional region, thereby promoting tissue growth (Cho *et al*., 2006; Feng *et al*., 2007). We thus performed qPCR analysis to investigate transcriptional levels of some pivotal genes in the Hippo signaling pathway in the wing disc at the wandering‐stage, including the Hippo upstream inputs *Ds* and *Ft*, the Hippo kinase cassette genes *Hippo* (*Hpo*) (Pan *et al*., 2007), *Warts* (*Wts*) (Justice *et al*., 1995) and *Mob‐as‐tumor‐suppressor* (*Mats*) (Lai *et al*., 2005), and the Hippo downstream transcriptional regulators *Yorkie* (*Yki*) (Huang *et al*., 2005), *Four‐jointed* (*Fj*) (Ishikawa *et al*., 2008), and *Wingless* (*Wg*) (Ko *et al*., 2016). The results revealed that transcriptional expression levels of *Ds* and *Ft* did not change in mutants, while *Hippo*, *Warts* and *Mats* were significantly up‐regulated and *Yki*, *Fj* and *Wg* were down‐regulated (Fig. 7), suggesting that *Bm‐app* depletion resulted in disturbance of the Hippo signaling pathway in *B. mori*.

## Discussion

In the current study, we identified that the silkworm *mw* phenotype in the u11 strain was attributed to down‐regulation of *Bm‐app* gene, caused by 10 bp insertion existing in the putative promoter region of *Bm‐app*. Furthermore, we confirmed this result by using the CRISPR/Cas9‐mediated targeted mutagenesis system. Microinjection of Cas9 mRNA and gene‐specific sgRNA elicited the *mw* phenotype in the Nistari moth with crooked small wings which were similar to that of the u11 mutant. *Bm‐app* is a homologous gene of palmitoyltransferase in *Drosophila*, which may regulate expression of related genes in the Hippo pathway to regulate the development of the wing disc.

Palmitoyltransferases are negative regulators of fat signaling in growth control (Linder & Deschenes, 2007; Feng & Irvine, 2009; Matis & Axelrod, 2013) and are characterized by the presence of a 50‐residue‐long domain called the DHHC domain (Politis *et al*., 2005; Drisdel *et al*., 2006), which in most but not all cases is also cysteine‐rich and gets its name from a highly conserved DHHC signature tetrapeptide (Asp‐His‐His‐Cys) (Nadolski & Linder, 2007; Fukata *et al*., 2016). In *Drosophila*, *App* encodes a four‐pass transmembrane protein containing a DHHC cysteine‐rich domain (Fukata & Fukata, 2010) and the catalytic domain in proteins function as palmitoyltransferase (Matakatsu & Blair, 2008). We performed qPCR analysis to investigate *Bm‐app* gene expression pattern in the wandering stage which is the important stage for wing disc development (Fig. S1). In this expression, *Bm‐App* had extensive expression in tissues. App binds both Ds and Ft and promotes the activity of Ds by simultaneously repressing Ft via post‐translational modification and recruiting Ds to the apical junctional region, thereby promoting tissue growth, including wing development (Cho *et al*., 2006; Matakatsu *et al*., 2017). Signaling via the large protocadherin Ft, regulated in part by its binding partner Ds and the Golgi‐resident kinase Four‐jointed (Fj), is required for a variety of developmental functions (Bennett & Harvey, 2006; Silva *et al*., 2006; Willecke *et al*., 2006).

Ds and Ft are important components of the Hippo signaling pathway which is a key regulator of organ size and is highly conserved in different organisms (Justice *et al*., 1995; Xu *et al*., 1995; Harvey *et al*., 2013). In *Drosophila*, the existence of the Hippo pathway coordinates multiple physiological processes, including developmental patterning, proliferation, apoptosis and cell growth, to regulate overall organ size (Boggiano & Fehon, 2012; Enderle & McNeill, 2013; Hariharan, 2015). In *B. mori*, all key components of the Hippo pathway exist (Liu *et al*., 2016; Zeng *et al*., 2017) and genes involved in the Hippo pathway are particularly enriched in several mitotic tissues, including the ovary, testis and wing disc in *B. mori* (Zeng *et al*., 2017; Cao *et al*., 2018). In the current study, although expressions of *Ds* and *Ft* did not show significant alteration, the Hippo kinase cassette genes *Hippo*, *Warts* and *Mats*, which play a part for inhibiting excessive cell proliferation, were significantly up‐regulated. In contrast, the Hippo downstream transcriptional regulators *Yki*, *Fj* and *Wg*, which are responsible for cell proliferation and anti‐apoptotic genes, were significantly down‐regulated. These genes involved in the Hippo pathway were affected after *Bm‐app* depletion, suggesting that *Bm‐app* may affect the Hippo pathway to regulate wing development in *B. mori*. We speculate that the down‐regulation of the Hippo pathway leads to minute wings (Zhang *et al*., 2017), which provides new insights into the role of Hippo pathway regulation in the silkworm. Furthermore, using the Cas9/sgRNA‐mediated mutagenesis provides a promising approach to explore molecular mechanisms of natural mutants in the future.

## Supporting information


**Table S1**. Primers used in this work.

**Table S2**. Summary of the 15 *mw* candidate genes in *B. mori*.

**Supplementary sequences**: putative promoter sequences of *Bm‐app*.

**Fig. S1**. The expression pattern of *Bm‐App* in wandering stage. Epi, epidermis; MG, midgut; FB, fat body; ASG, anterior silk gland; MSG, middle silk gland; PSG, posterior silk gland; TE, testes; OV, ovary; MT, Malpighian tubule; WD, wing disc.
