# Supplementary material for: A palmitoyltransferase Approximated gene Bm‐app regulates wing development in Bombyx mori
Source: Insect Sci. 2018 Aug 23;27(1):2–13. doi: 10.1111/1744-7917.12629 (PMC7379679; doi:10.1111/1744-7917.12629)
Supplement: Supplementary file 1 — Table S1. Primers used in this work. [file INS-27-2-s001.docx]

Table S1 Primers used in this work

| Primer name | Primer sequence(5’to3’) | The purpose |
| --- | --- | --- |
| C17-F | GGATGGAGGTGGCTCAATAG | Q-RT-PCR |
| C17-R | GGCTGCGATACACGAGGAAT | Q-RT-PCR |
| C18-F | CAAATCCCATCTGGCTGAA | Q-RT-PCR |
| C18-R | CTCCACGCCATAATGATTCTAC | Q-RT-PCR |
| C19-F | ATCCGCCCTTTACTATTCCA | Q-RT-PCR |
| C19-R | TGCCCAGTATGTCCTGTCTATG | Q-RT-PCR |
| C20-F | TGGAAACACGGGTGTATGG | Q-RT-PCR |
| C20-R | AATGTTACTGACCTCGCCTGA | Q-RT-PCR |
| C21-F | TGGTAAACACCGACAGTAAT | Q-RT-PCR |
| C21-R | GCCCTAAAGTTCTGGTGGTA | Q-RT-PCR |
| C22-F | GTTTTTGGCGTCTTCCATGG | Q-RT-PCR |
| C22-R | ATTTCTCTATCGATAGGTACC | Q-RT-PCR |
| C23-F | CGAGCCACACTACTACATCG | Q-RT-PCR |
| C23-R | TGCCACAGAATGTCACGATG | Q-RT-PCR |
| C24-F | AGGATCTCTCTGCACACGG | Q-RT-PCR |
| C24-R | CGCCGTCTTTAGTCTTGTCG | Q-RT-PCR |
| C25-F | AATAGTTGGCTTCCTGTGCG | Q-RT-PCR |
| C25-R | ACACGGGTCCAATAGTTCGT | Q-RT-PCR |
| C26-F | GCAGACGAACCCTTCAATCC | Q-RT-PCR |
| C26-R | CATAACGTTTCGACCGCCAT | Q-RT-PCR |
| C27-F | ACATGACACTGCTAACCCCA | Q-RT-PCR |
| C27-R | TCTGGATGTGGAGACGGAAG | Q-RT-PCR |
| C51-F | AGGTAATTCCAATAGTCTAG | Q-RT-PCR |
| C51-R | CTAGACTATTGGAATTACCT | Q-RT-PCR |
| C52-F | GACGCCCTCGCTGATCGATA | Q-RT-PCR |
| C52-R | TATCGATCAGCGAGGGCGTC | Q-RT-PCR |
| C53-F | ATCTTCCTCCGCGAGTTGAT | Q-RT-PCR |
| C53-R | ATCTTCCTCCGCGAGTTGAT | Q-RT-PCR |
| C54-F | CGCAAGTGGGAAGTGTTCG | Q-RT-PCR |
| C54-R | AGGAAGGGACAGTCGAACG | Q-RT-PCR |
| app-sg1-F | TAATACGACTCACTATAGGCGCCCACGGCGGGCACGGGTTTTAGAGCTAGAAATAGCAAGTTAAAATAAGGCTAGTCC | sgRNA construction |
| app-sg2-F | TAATACGACTCACTATAGGGGGGAAGCCACCACCGACGTTTTAGAGCTAGAAATAGCAAGTTAAAATAAGGCTAGTCC | sgRNA construction |
| sgRNA-R | AAAAGCACCGACTCGGTGCCACTTTTTCAAGTTGATAACGGACTAGCCTTATTTTAACTTGCTATTTCTAGCTCTAAAA | sgRNA construction |
| Primer1-F | GCAGGACATGGTGCGATG | Mutation detection |
| Primer2-R | GCAGGACATGGTGCGATG | Mutation detection |
| Promoter-F | CGGGGTACC CGAGCCACACTACTACATCG | Plasmid construction |
| Promoter-R | CCCAAGCTT CGTTCCCAACTCACGTTTCA | Plasmid construction |
| *BmHippo*-F | GGACCTGTGGTCCCTATG | Q-RT-PCR |
| *BmHippo*-R | CGCTGTCCGAACCATGGA | Q-RT-PCR |
| *BmWarts*-F | CTTCACGGACACAGAAACGA | Q-RT-PCR |
| *BmWarts*-R | CTCACCAAACGAGGGACATT | Q-RT-PCR |
| *BmMats*-F | CAGGGAATTCGTTGATACCG | Q-RT-PCR |
| *BmMats*-R | TCATCCAGCAAGTCTTCACG | Q-RT-PCR |
| *BmYki*-F | CAGAATGGCGGTACTTGTCG | Q-RT-PCR |
| *BmYki*-R | CGACACGTGGCTCCATTAAG | Q-RT-PCR |
| *BmFj*-F | CCTCTTCGACTTGTGGCTTG | Q-RT-PCR |
| *BmFj*-R | TTGCCGTTTTCTGATTGCCA | Q-RT-PCR |
| *BmWg*-F | AACGGGACAGCAATGAGAGA | Q-RT-PCR |
| *BmWg*-R | TCCAGTGTAAGGGTCGCATT | Q-RT-PCR |
| *BmDs*-F | AGATTGGCCAGAGAGAACCC | Q-RT-PCR |
| *BmDs*-R | ACGGCAACCTCTATCCACAA | Q-RT-PCR |
| *BmFt*-F | AAGTCATGGTGGCAATCAGA | Q-RT-PCR |
| *BmFt*-R | GAATAATAAAATTGCCGGTAC | Q-RT-PCR |
