# Supplementary material for: A palmitoyltransferase Approximated gene Bm‐app regulates wing development in Bombyx mori
Source: Insect Sci. 2018 Aug 23;27(1):2–13. doi: 10.1111/1744-7917.12629 (PMC7379679; doi:10.1111/1744-7917.12629)
Supplement: Supplementary file 2 — Table S2. Summary of the 15 mw candidate genes in B. mori. [file INS-27-2-s002.docx]

Table S2. Summary of the 15 *mw* candidate genes in *B. mori*.

| No. | SilkDB ID | Description |
| --- | --- | --- |
| C17 | BGIBMGA012717 | transposable elements^1^ |
| C18 | BGIBMGA012718 | triose-phosphate transporter |
| C19 | BGIBMGA012719 | No |
| C20 | BGIBMGA012720 | vestigial |
| C21 | BGIBMGA012721 | No |
| C22 | BGIBMGA012722 | carboxylesterase CarE-8 variant 1 |
| C23 | BGIBMGA012723 | No |
| C24 | BGIBMGA012724 | reverse transcriptase/integrase |
| C25 | BGIBMGA012725 | No |
| C26 | BGIBMGA012726 | neurofilament heavy polypeptide |
| C27 | BGIBMGA012727 | chemosensory ionotropic receptor |
| C51 | BGIBMGA012751 | peroxidasin-like protein |
| C52 | BGIBMGA012752 | carboxyl/choline esterase |
| C53 | BGIBMGA012753 | Endoplasmin-like |
| C54 | BGIBMGA012754 | palmitoyltransferase app |
