# Supplementary material for: A palmitoyltransferase Approximated gene Bm‐app regulates wing development in Bombyx mori
Source: Insect Sci. 2018 Aug 23;27(1):2–13. doi: 10.1111/1744-7917.12629 (PMC7379679; doi:10.1111/1744-7917.12629)
Supplement: Supplementary file 3 — Supplementary sequences: putative promoter sequences of Bm‐app. [file INS-27-2-s003.docx]

**Supplementary sequences: putative promoter sequences of *Bm-app.***

>putative promoter sequence in p50 strain

TTGAGTGTGCACCGCACATGACCTTTAGACATTAAAGTATATTAGCTAGCTATTATGTAATGTATATTTATTCCAGATTACTAATCTTTAATAAATATGATGCTGACTAAACTTTTTTATTGCACTAAGGAAACAGCCTACTCTTTAGTGATCTGTGCCTTATAGATCTGCTTCGCCAGAGGCATACTAAAGCTACTGCCTACTGTTTTGAGAAAATCATGCTTAACTTTTCTTTTTCTTGTCCATTTATGTATACATAAGTTTGATAATATAATATTTAATATTTAGTGACTGCACCAGCCTATTTATTTGTGAAAATATACACTTATCCTAATATATTTTGAAAAACAAATTCTCCTAGCCCATTAAATATAAGTTTTATCACAATTTCAAGTAAAAGTTGATAGTCAAACTACTTATAATAAAAAAAATCCCTTTACAGTTTTTTTAATTTATGGGACGGAAGTTAACATAAATTCGGAAGACACACTTTACCTAGTTCACTCCTATCGAGCCACACTACTACATCGTGACATTCTGTGGCAGTATTATATGTAGGTAAATACTTTCTAATCACTATTAACACATCGACGGCCCCTACATACAAAGCTTCATGTCAAAAATGGTGTTTTACAGGAGAGTTTACTGGTGGTAGGACCTCTTGTGAGTCTGCGCGGGTGGGTACCACCTCCCTGCCTATTTCTACCGTGAAGGTTTTGTTTGTTGTCAATAGATGGCGCGGTATATATTTCTAAAACGCGCTATAGACATGTGACAAAGAGAAATACAAAAACGTTTCGTCACATTTGCGGAGATATATAAGTGAACACAAATGTTTTAGTTACATATTACTAGAAGTTTTAGGCGGTTCGCATGACAAGGTGCAATGCCTGATTAAATTAATACAAATCATGATGCGCATGCAGTGTAACGTCAGTTTACGTCACGCCACGCGCTTATTCACAAACACTACACAAGCGCAACGTGTGAATGTGTTGAACGCGAGCTACATAGTAGGCGTGAGATATATAAGTGAACACAAATGTTTTAGTTACATATTACTAGAATCGCCGCGCTCATGGCCCGCAATAAAATCTATGCAATAGCTTAATAATTTTTTAAACCGTATAAAATATAAATGTTCACGTAAAGGTATCTACATCAACAGTCTTTTTATTTTTAAAAGTTGACATCTTATACGTTTATCTTTAAAACGTTTACTTATAAAATAAAAAAAATTATATGGTAATGTATGTGTTGACAAATAGAAAATGATTATGAAATAATAATAAAACGTAAACGTAAAAATAAAACTTTATATGTAGGGACCGTCGACATGCTCATTAATAACTAATAGGATAAAGGAATAGTACTGTCTTAAACATATGCTCATATAACTTATAACTTTGCCTAAGTATTAGAGGTAGGGTGTGTTATGAACTGCTATAAACAGTTTTACCACCCTGATTAAGATTGCGTTGATGGCTCTGTTGTCCATTTCATACTATTAATTTTATAATTCATAAAACCTATAACATCTCATAAGACAAGGGCCGTCTATAATTTGACCACAAATAATAATTTTAATAAAAATGTTAATTTTCTTCCTTGAATCAGGACATGTCCTTCTTCTAAGGATCATTAGGAAAAATAATCAATTAAATGATAGAAATAAACAAGAATTATTTTAACTTTCTGTATACTAAGTAGCAATTTCATTGGTGAATACTTAATATTATACCCTTTGTCGCTTCCACAACTCAGACGAATCCTATGATATCTCATATATTAAAATCATCTAAAAGGTGTAGAGCATCAGTCAACAAAGACACCCCTGAAAAACAATATCCGTCTTCGGTAGTCCGGTAAAGAACTTATAGCCCTGTGCACTCGAGCATTGTAGATCCGTATGTTTCCATCACATTTAAATAAATACAATTATTATATAGGCTGAAACGTGAGTTGGGAACGCGAGAGTGGGAGCGGCAGGACATGGTGCGATG

>putative promoter sequence in u11 strain

TTGAGTGTGCACCGCACATGACCTTTAGACATTAAAGTATATTAGCTAGCTATTATGTAATGTATATTTATTCCAGATTACTAATCTTTAATAAATATGATGCTGACTAAACTTTTTTATTGCACTAAGGAAACAGCCTACTCTTTAGTGATCTGTGCCTTATAGATCTGCTTCGCCAGAGGCATACTAAAGCTACTGCCTACTGTTTTGAGAAAATCATGCTTAACTTTTCTTTTTCTTGTCCATTTATGTATACATAAGTTTGATAATATAATATTTAATATTTAGTGACTGCACCAGCCTATTTATTTGTGAAAATATACACTTATCCTAATATATTTTGAAAAACAAATTCTCCTAGCCCATTAAATATAAGTTTTATCACAATTTCAAGTAAAAGTTGATAGTCAAACTACTTATAATAAAAAAAATCCCTTTACAGTTTTTTTAATTTATGGGACGGAAGTTAACATAAATTCGGAAGACACACTTTACCTAGTTCACTCCTATCGAGCCACACTACTACATCGTGACATTCTGTGGCAGTATTATATGTAGGTAAATACTTTCTAATCACTATTAACACATCGACGGCCCCTACATACAAAGCTTCATGTCAAAAATGGTGTTTTACAGGAGAGTTTACTGGTGGTAGGACCTCTTGTGAGTCTGCGCGGGTGGGTACCACCTCCCTGCCTATTTCTACCGTGAAGGTTTTGTTTGTTGTCAATAGATGGCGCGGTATATATTTCTAAAACGCGCTATAGACATGTGACAAAGAGAAATACAAAAACGTTTCGTCACATTTGCGGAGATATATAAGTGAACACAAATGTTTTAGTTACATATTACTAGAAGTTTTAGGCGGTTCGCATGACAAGGTGCAATGCCTGATTAAATTAATACAAATCATGATGCGCATGCAGTGTAACGTCAGTTTACGTCACGCCACGCGCTTATTCACAAACA

　　　　　　　　　　　　　　　　　　　　　　　　　　　　　　　ACATAGTAGT

CTACACAAGCGCAACGTGTGAATGTGTTGAACGCGAGCTACATAGTAGGCGTGAGATATATAAGTGAACACAAATGTTTTAGTTACATATTACTAGAATCGCCGCGCTCATGGCCCGCAATAAAATCTATGCAATAGCTTAATAATTTTTTAAACCGTATAAAATATAAATGTTCACGTAAAGGTATCTACATCAACAGTCTTTTTATTTTTAAAAGTTGACATCTTATACGTTTATCTTTAAAACGTTTACTTATAAAATAAAAAAAATTATATGGTAATGTATGTGTTGACAAATAGAAAATGATTATGAAATAATAATAAAACGTAAACGTAAAAATAAAACTTTATATGTAGGGACCGTCGACATGCTCATTAATAACTAATAGGATAAAGGAATAGTACTGTCTTAAACATATGCTCATATAACTTATAACTTTGCCTAAGTATTAGAGGTAGGGTGTGTTATGAACTGCTATAAACAGTTTTACCACCCTGATTAAGATTGCGTTGATGGCTCTGTTGTCCATTTCATACTATTAATTTTATAATTCATAAAACCTATAACATCTCATAAGACAAGGGCCGTCTATAATTTGACCACAAATAATAATTTTAATAAAAATGTTAATTTTCTTCCTTGAATCAGGACATGTCCTTCTTCTAAGGATCATTAGGAAAAATAATCAATTAAATGATAGAAATAAACAAGAATTATTTTAACTTTCTGTATACTAAGTAGCAATTTCATTGGTGAATACTTAATATTATACCCTTTGTCGCTTCCACAACTCAGACGAATCCTATGATATCTCATATATTAAAATCATCTAAAAGGTGTAGAGCATCAGTCAACAAAGACACCCCTGAAAAACAATATCCGTCTTCGGTAGTCCGGTAAAGAACTTATAGCCCTGTGCACTCGAGCATTGTAGATCCGTATGTTTCCATCACATTTAAATAAATACAATTATTATATAGGCTGAAACGTGAGTTGGGAACGCGAGAGTGGGAGCGGCAGGACATGGTGCGATG

Sequences in blue indicate the putative promoter sequences predicted by the online software in FlyBase (http://www.fruitfly.org/seq_tools/promoter.html). The score cutoff was set as 0.8. Black arrow indicates the position of insertion. Start codon ATG was in green and 10-bp insertion sequence (ACATAGTAGT) was in red and indicated by the arrow. Six predicted promoter sequences with score cutoff 0.80 (transcription start shown in larger font) were listed below:

| Start | End | Score | Predicted promoter sequences |
| --- | --- | --- | --- |
| 735 | 785 | 0.96 | TGGCGCGGTATATATTTCTAAAACGCGCTATAGACATGTGACAAAGAGAA |
| 808 | 858 | 0.82 | TGCGGAGATATATAAGTGAACACAAATGTTTTAGTTACATATTACTAGAA |
| 1018 | 1068 | 0.80 | GCGTGAGATATATAAGTGAACACAAATGTTTTAGTTACATATTACTAGAA |
| 1124 | 1174 | 1.00 | CCGTATAAAATATAAATGTTCACGTAAAGGTATCTACATCAACAGTCTTT |
| 1306 | 1356 | 0.96 | AAATAAAACTTTATATGTAGGGACCGTCGACATGCTCATTAATAACTAAT |
| 1549 | 1599 | 0.89 | AAGGGCCGTCTATAATTTGACCACAAATAATAATTTTAATAAAAATGTTA |
