# Supplementary material for: A palmitoyltransferase Approximated gene Bm‐app regulates wing development in Bombyx mori
Source: Insect Sci. 2018 Aug 23;27(1):2–13. doi: 10.1111/1744-7917.12629 (PMC7379679; doi:10.1111/1744-7917.12629)
Supplement: Supplementary file 4 — Fig. S1. The expression pattern of Bm‐App in wandering stage. Epi, epidermis; MG, midgut; FB, fat body; ASG, anterior silk gland; MSG, middle silk gland; PSG, posterior silk gland; TE, testes; OV, ovary; MT, Malpighian tubule; WD, wing disc. [file INS-27-2-s004.docx]

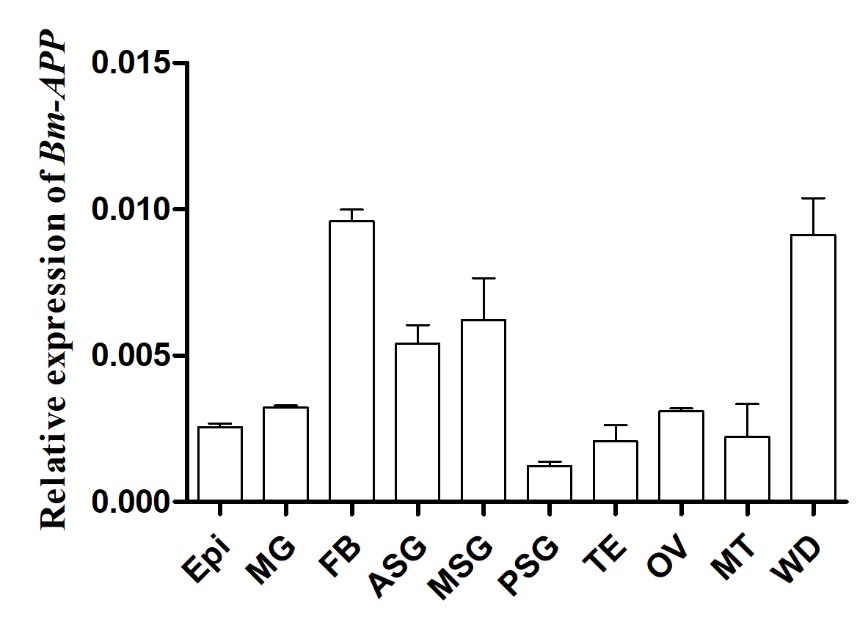


**Fig. S1. The expression pattern of *Bm-App* in wandering stage.** Epi, epidermis; MG, midgut; FB, fat body; ASG, anterior silk gland; MSG, middle silk gland; PSG, posterior silk gland; TE, testes; OV, ovary; MT, malpighian tubule; WD, wing disc.

As for the only mutation in wing disc, we speculated that *Bm-App* had not play an important role in other tissues, although it had high expression in some tissues.
